# Uncovering production of specialized metabolites by *Streptomyces argillaceus*: Activation of cryptic biosynthesis gene clusters using nutritional and genetic approaches

**DOI:** 10.1371/journal.pone.0198145

**Published:** 2018-05-24

**Authors:** Adriana Becerril, Susana Álvarez, Alfredo F. Braña, Sergio Rico, Margarita Díaz, Ramón I. Santamaría, José A. Salas, Carmen Méndez

**Affiliations:** 1 Departamento de Biología Funcional e Instituto Universitario de Oncología del Principado de Asturias (I.U.O.P.A), Universidad de Oviedo, Oviedo, Spain; 2 Instituto de Investigación Sanitaria de Asturias (ISPA), Oviedo, Spain; 3 Departamento de Microbiología y Genética, Instituto de Biología Funcional y Genómica, Consejo Superior de Investigaciones Científicas (CSIC)/Universidad de Salamanca, Salamanca, Spain; Universite Paris-Sud, FRANCE

## Abstract

Sequencing of *Streptomyces* genomes has revealed they harbor a high number of biosynthesis gene cluster (BGC), which uncovered their enormous potentiality to encode specialized metabolites. However, these metabolites are not usually produced under standard laboratory conditions. In this manuscript we report the activation of BGCs for antimycins, carotenoids, germicidins and desferrioxamine compounds in *Streptomyces argillaceus*, and the identification of the encoded compounds. This was achieved by following different strategies, including changing the growth conditions, heterologous expression of the cluster and inactivating the *adpAa* or overexpressing the *abrC3* global regulatory genes. In addition, three new carotenoid compounds have been identified.

## Introduction

*Streptomyces* are Gram-positive bacteria with high GC content that show a differentiation cycle. They are known for being a prolific source of secondary metabolites (also referred here as specialized metabolites) [1], many of which show some kind of bioactivity [2]; for example, about 39% of bioactive compounds produced by microorganisms have a *Streptomyces* origin [3]. Traditionally, any *Streptomyces* strain was known to produce only one or a few specialized metabolites. However, since the first *Streptomyces* genome was sequenced it was brought to light that they harbored a higher number of biosynthesis gene clusters (BGC) than expected, which untapped their enormous potential to synthesize bioactive compounds [4]. During the last years, due to the development of next-generation sequencing technologies and bioinformatics tools such as AntiSMASH [5] and MIBiG [6] to identify candidate BGCs, a huge number of *Streptomyces* genome sequences has been made public, confirming their potential for synthesizing specialized metabolites [4,7]. Curiously, most of these metabolites are not usually identified under standard laboratory growth conditions even those compounds encoded by BGCs frequently found in *Streptomyces* genomes, which indicate that many of these BGCs are poorly or not expressed under those conditions. Therefore, several strategies have been developed to activate these silent BGCs and to identify the encoded compounds, which involves either genetic and/or culture conditions interventions [7–9].

*Streptomyces argillaceus* ATCC 12956 is known for producing the antitumor mithramycin, whose biosynthesis gene cluster has been cloned and thoroughly characterized [10]. However, no other specialized metabolite had been described in this strain until very recently, when its genome was sequenced and mined [11]. This has led to the identification of 31 putative BGCs for specialized metabolites none of them previously detected under standard laboratory growth conditions. Characterization of one of these clusters (cluster *arp*) led to the discovery of a new family of polyketide alkaloids named argimycins P [11,12]. Among the other identified BGCs, two were highly conserved and proposed to be involved in the biosynthesis of antimycins (cluster 27) and isorenieratene (cluster 26). However, their encoded compounds have not been identified so far in cultures of this strain, which suggests these BGCs are silenced or not normally expressed under standard laboratory culture conditions. Herein we report the use of nutritional and genetic approaches to activate expression of those BGCs in order to identify their encoded compounds. In addition, we report that inactivation of a global regulatory gene (an *adpA*-like) and overexpression of the *S*. *coelicolor* response regulator *abrC3* led to the production of germicidins and to activate/increase production of several metabolites including the specialized metabolite desferrioxamine, respectively.

## Material and methods

### Strains, culture conditions, plasmids and DNA manipulations

*S*. *argillaceus* ATCC 12956, a mithramycin producer, was used as source of DNA. *S*. *argillaceus* and *S*. *albus* J1074 (ilv-1, sal-2) [13] were used as hosts for gene expression. R5A [14], SM17 [11], SM3, SM4, SM19, SM30 and SV2 media (S1 File) were used for production of secondary metabolites, following a two-step culture method [14]. *Escherichia coli* ET12567/pUB307 was used as donor in conjugation experiments [13]. When required, antibiotics were used at the following final concentrations: apramycin (25 μg/ml), thiostrepton (50 μg/ml), ampicillin (100 μg/ml), kanamycin (50 μg/ml), and nalidixic acid (25 μg/ml). A pKC505-based cosmid library of *S*. *argillaceus* chromosomal DNA [15] was used to identify cosmids containing the *crta* gene cluster, by using two DNA fragments obtained by PCR amplification using oligonucleotides genot25I-up and genot25I-rp (fragment I), and genot25D-up and genot25D-rp (fragment D) (S1 Table). DNA manipulations and transformations/conjugations were carried out using standard procedures [13,16]. Sequences analyses were performed using antiSMASH 4.1.0 [5] and BLAST [17]. Limits of each cluster were proposed after comparing genes (and their gene products) located at both ends with others in data-bases.

### Plasmid constructs

pHJLAbrC3c was constructed to express the *abrC3* gene from *S*. *coelicolor* [18] as follows: a PvuII DNA fragment containing the *oriT* and the apramycin resistance cassette was obtained from pIJ773 [19] and subcloned into the EcoRV of pHJLAbrC3 [18].

pEM4T-sigmaC25 was generated to express *crtaQ* as follows: a 697 bp DNA fragment containing the *crtaQ* gene was amplified from cosmid pKC505-C25 as a BamHI-EcoRI fragment using oligonucleotides SigmaC25up and Sigma C25rp (S1 Table), and cloned into the same sites of pEM4T [20].

pΔadpAa was constructed to generate mutant *S*. *argillaceus* Δ*adpAa* as follows: a 1.8 kb DNA fragment containing the 3’-end of *adpAa* and downstream DNA region, was amplified using oligonucleotides adpA-5BglII and adpA-2R (S1 Table), digested with BglII and EcoRV, and subcloned into BamHI and EcoRV sites of pEFBAoriT [21], generating pEFBAadpAa2. Also, a 2.5 kb DNA fragment containing the 5’-end of *adpAa* was amplified using oligonucleotides adpA-3SpeI and adpA-3NsiI (S1 Table), digested with SpeI and NsiI and subcloned into the same sites of pEFBAadpAa2, upstream of the apramycin resistance cassette, generating pEFBAadpAa21. Finally, a hygromycin resistance cassette was isolated as a SpeI-NheI fragment from pLHyg [22] and subcloned into the XbaI site of pEFBAadpAa21.

### Extraction and analysis of secondary metabolites

Culture samples (1 ml) were extracted with 1 volume of ethyl acetate and 1% formic acid (antimycins), *n*-butanol (desferrioxamine and germicidins) or methanol: tetrahydrofuran (THF) 1:1 and 1% of butylated hydroxytoluene (BHT) (carotenoid compounds). The organic extracts were evaporated *in vacuo* and dissolved in a small volume of DMSO:methanol (50:50). Samples were analyzed by reversed-phase chromatography on Acquity UPLC equipment with a BEH C18 column (1.7 mm, 2.1 x 100 mm; Waters, Milford, MA, USA), using acetonitrile and 0.1% trifluoroacetic acid (TFA) in water as eluent. Samples were eluted with 10% acetonitrile for 1 min, followed by a linear gradient from 10 to 90% acetonitrile over 7 min at a flow rate of 0.5 ml/min and a column temperature of 35°C. For the carotenoid compounds, sample analyses were performed by reversed-phase chromatography on Alliance HPLC equipment with a SunFire C18 column (3.5 μm, 2.1 x 150 mm, Waters, Milford, MA, USA), using a mixture of methanol:acetonitrile (65:35) as eluent in isocratic conditions for 20 min, at a flow rate of 0.25 ml/min and a column temperature of 35°C. Detection and spectral characterization of peaks were carried out with a photodiode array detector and Empower software (Waters). Mass analysis was performed by reversed-phase chromatography on Alliance HPLC equipment, coupled to a ZQ4000 mass spectrometer, with a SunFire C18 column (3.5 μm, 2.1 x 150 mm, Waters, Milford, MA, USA) with acetonitrile and 0.1% trifluoroacetic acid (TFA) in water as eluent. Samples were eluted with 10% acetonitrile for 4 min, followed by a linear gradient from 10 to 90% acetonitrile over 26 min at a flow rate of 0.25 ml/min and a column temperature of 35°C. Detection and spectral characterization of peaks were carried out with a photodiode array detector and Empower software (Waters). Mass analyses were carried out as previously reported [23]. The identity of compounds was determined by dereplicating the extracts by LC-UV-LRMS and LC-HMRS against The Chapman & Hall Dictionary of Natural Products [24] and a database from Fundación Medina [25].

### Purification of germicidins

*S*. *argillaceus* Δ*adpAa* was cultivated in five 2-liter Erlenmeyer flasks, each containing 400 ml of R5A medium, at 30°C for five days. The cultures were centrifuged and the pellets were discarded. The supernatants were filtered, acidified by adding formic acid up to 1% and applied to a solid-phase extraction cartridge (Sep-Pak Vac C18, 10g, Waters). The retained material was eluted with a mixture of methanol and 0.05% TFA in water. A linear gradient from 0 to 100% methanol in 60 min, at 10 ml/min, was used. Analysis of fractions taken every 5 min indicated that de desired compounds eluted between 30 and 40 minutes. The corresponding fractions were pooled, evaporated *in vacuo* and redissolved in 2 ml of DMSO. The germicidins were purified by preparative HPLC using a SunFire C18 column (10 μm, 10 x 250 mm, Waters). The extract was chromatographed in isocratic conditions, first with a mixture of acetonitrile and 0.1% TFA in water (55:45), and subsequently with a mixture of methanol and 0.1% TFA in water (75:25), at 5 ml/min. After every purification step, the collected material was diluted fourfold with water and then applied to a solid-phase extraction cartridge (Sep-Pak C18, Waters). The cartridge was washed with water, and the retained compounds were eluted with methanol and dried *in vacuo*. Once the purification was finished, the purified material was dissolved in a mixture of tert-butanol and water (1:1) and lyophilized, with a resulting yield of 5.2 mg.

### Nucleotide sequences

Nucleotide sequences of gene clusters were deposited at the European Nucleotide Archive (EBI-ENA) under the accession numbers LT989885 (*anta*), LT989886 (*crta*), LT989884 (*gcsA*) and LT989883 (*desa*), and at Minimum Information about a Biosynthetic Gene Cluster (MIBIG) [6] under the accession numbers BGC0001455 (*anta*), BGC0001456 (*crta*), BGC0001454 (*gcsA*) and BGC0001453 (*desa*). The *adpAa* DNA region has been deposit at EBI-ANA under the accession number LS398138.

## Results and discussion

### Growth of *S*. *argillaceus* in different culture media resulted in activation of antimycins biosynthesis gene cluster 27 (*anta*)

**Fig 1:**
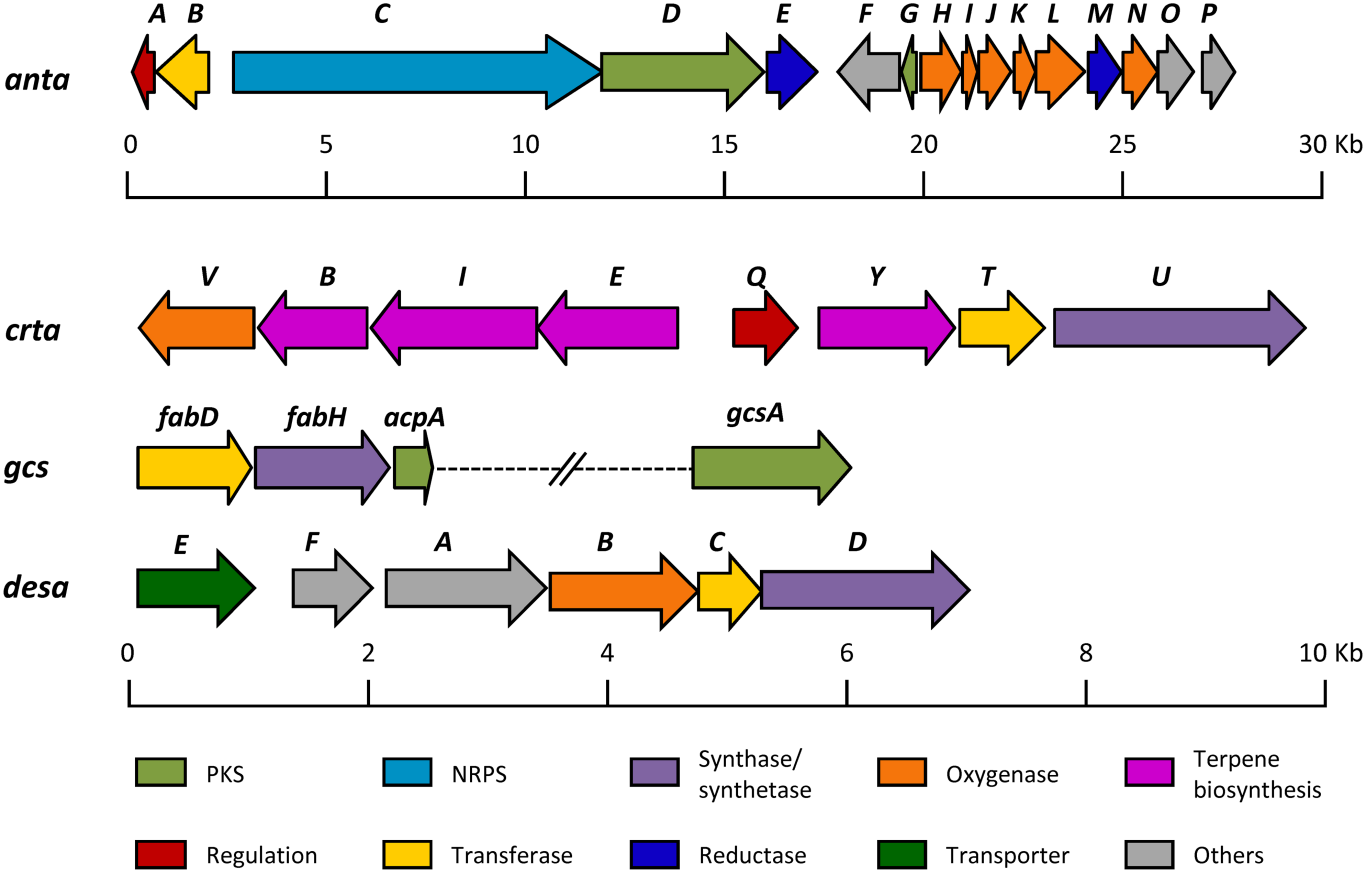
Gene organization of antimycins (*anta*), isorenieratene (*crta*), germicidines (*gcsA*) and desferrioxamine (*desa*) biosynthesis gene clusters in *S*. *argillaceus*. Genes are shown to scale. Information about gene functions is shown in S2 to S5 Tables.

Cluster 27 (*anta*; nucleotides 7920176–7946926 (Fig 1) shows high similarity to antimycins BGCs (S2 Table).

**Fig 2:**
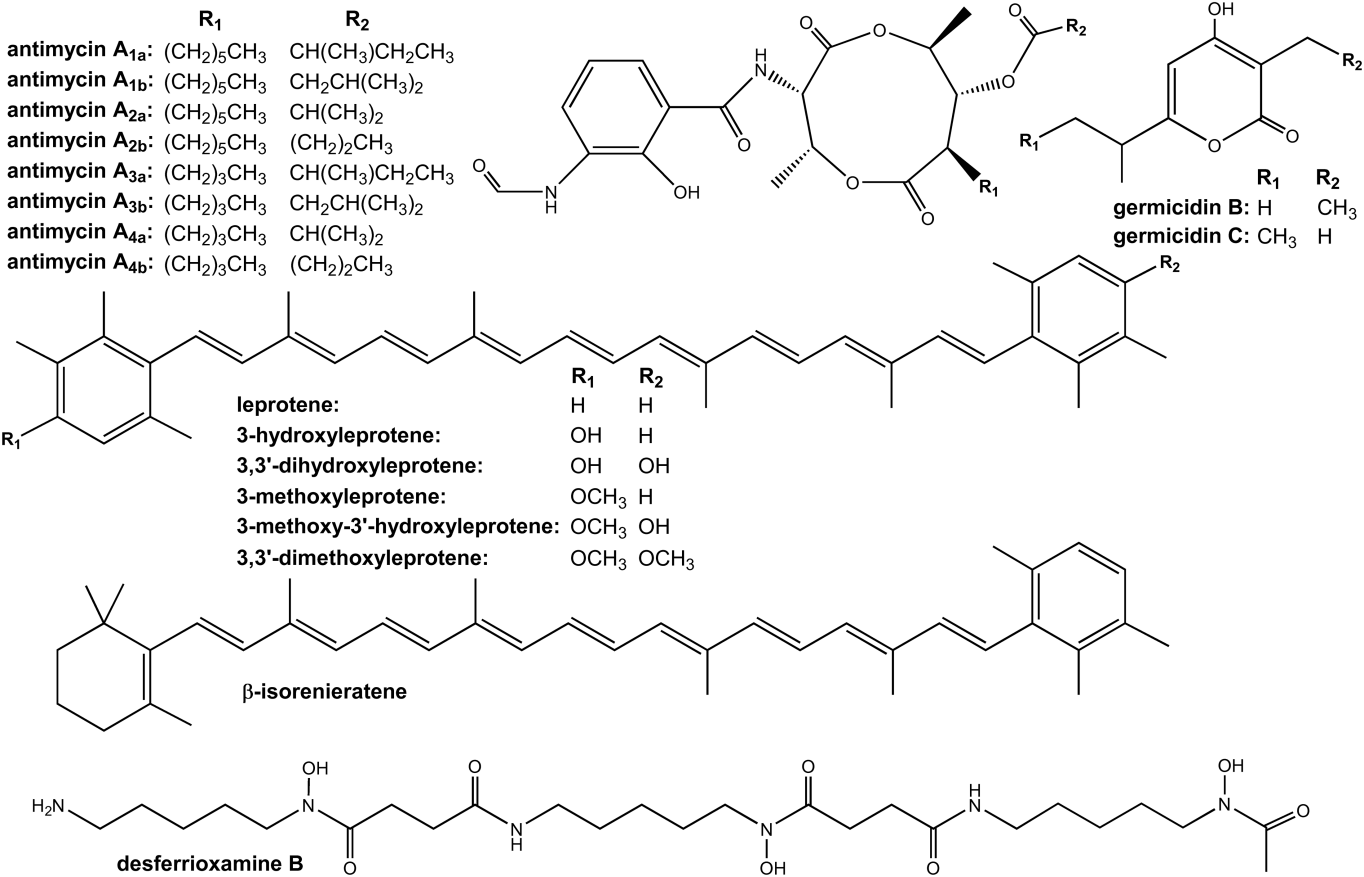
Chemical structures of compounds identified in *S*. *argillaceus* cultures in this work.

Antimycins are bioactive compounds characterized by a 9-membered dilactone core synthesized by a PKS-NRPS system bearing variations at C7 and C8, which is connected by an amide linkage to a 3-formamidosalicylic acid moiety [26] (Fig 2).

Antimycins BGCs are widespread among *Actinobacteria*: 73 antimycins BGC were identified in a recent study analyzing 1421 public genomes from selected genera of *Actinobacteria* [27]. All antimycins BGC identified so far show high synteny among them, and according to the number of genes and the presence/absence of the kynureninase *antP* and the phosphopantetheinyl transferase *antQ* they have been classified into four different classes: long-form (L-form), intermediate-form (I_P_- or I_Q_-form) and short-form (S-form) [26,27]. The *anta* cluster contains all genes required for the biosynthesis of antimycins (Fig 1; S2 Table): biosynthesis and activation of 3-formamidosalicylate starter unit (*antaFGHIJKLNO*); biosynthesis and modification of the 9-membered dilactone core (*antaBCDEM*); and regulation (*antaA*). Since it lacks the *antQ*-like but contains an *antP*-like gene, the *anta* cluster can be classified as an I_P_-form class.

**Fig 3:**
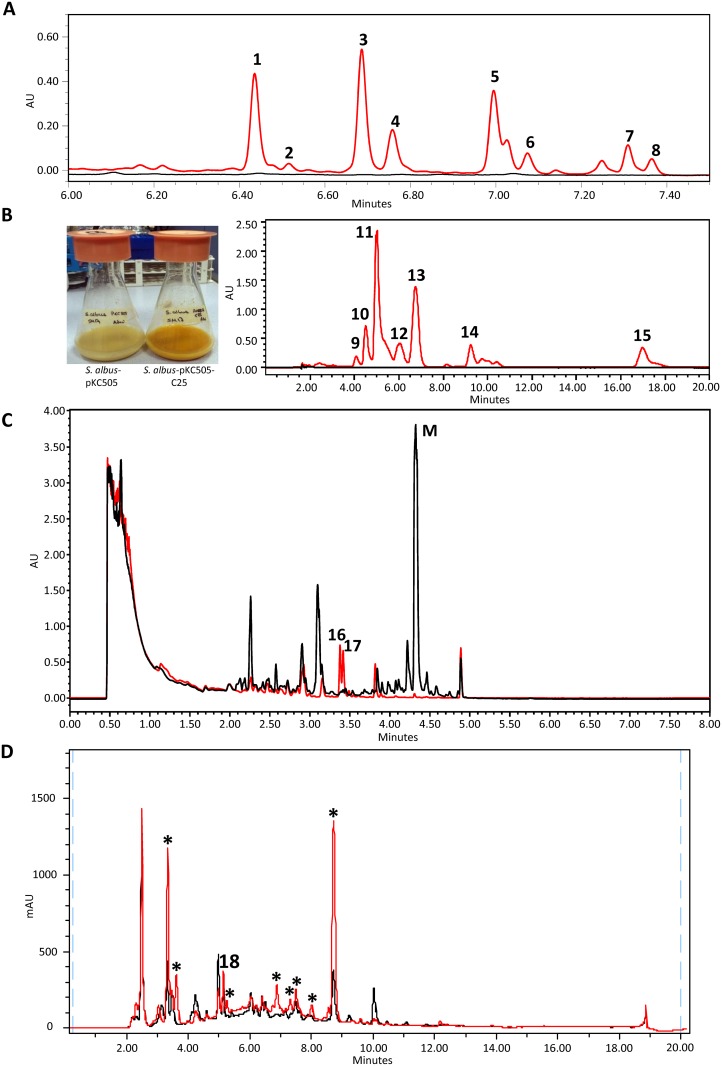
Production of specialized metabolites by *S*. *argillaceus*. (A) antimycins (peaks **1** to **8**): UPLC chromatogram at 230 nm of *S*. *argillaceus* grown in R5A (black) and in SM30 (red) media; (B) carotenoids: (left) Erlenmeyer flasks containing cultures of *S*. *albus*-pKC505 and *S*. *albus*-pKC505-C25, and (right) HPLC chromatograms at 450 nm showing production of carotenoids (peaks **9** to **15**) of *S*. *albus*-pKC505 (black) and *S*. *albus*-pKC505-C25 (red) in SM17; (C) germicidins (peaks **16** and **17**): UPLC chromatogram at 290 nm of *S*. *argillaceus* Δ*adpAa* (red) and *S*. *argillaceus* wild type (black). **M**, mithramycin; (D) desferrioxamine B (peak **18**): HPLC chromatogram at 210 nm of *S*. *argillaceus*-pHJLAbrC3c (red) and *S*. *argillaceus*-pHJL401c (black). Asterisks correspond to increased production of unknown compounds.

Antimycins possess a characteristic absorption spectrum with maxima around 231 and 317 nm. However, no peaks with this spectrum were identified in *S*. *argillaceus* cultures in R5A medium (standard laboratory conditions). Since production of specialized metabolites is greatly influenced by changing culture conditions [28], production of antimycins was evaluated in a set of different media. This resulted in detection of antimycins production in five (SM3, SM4, SM19, SM30 and SV2) out of twenty five different media tested: four different pair of peaks were detected with the characteristic absorption spectrum of antimycins (peaks **1** to **8**, Fig 3A), and with *m/z* values in positive mode of 507, 521, 535 and 549 (S1 Fig) that fit with the corresponding masses for antimycin A_4a/4b_, A_3a/3b_, A_2a/2b_ and A_1a/1b_, respectively (Fig 2). Moreover, these antimycins showed the same retention times than antimycins A_1_-A_4_ previously identified in *S*. *albus* [29], confirming the identity of these compounds.

### Activation of isorenieratene biosynthesis gene cluster 26 (*crta*) by its expression in a heterologous host

Cluster 26 (*crta*; nucleotides 7573410–7583894) shows high similarity to carotenoid isorenieratene BGCs (Fig 1; S3 Table). Carotenoids are terpenoids found in all photosynthetic organisms and also in some nonphototrophic organisms. They have different applications as food colorants, feed supplements, nutraceuticals and pharmaceuticals [30]. Many streptomycete strains contain isorenieratene BGCs that are usually silent. Only in a few cases activation of these BGCs has been achieved and production of their encoded compounds proved experimentally [31–33]. The *crta* cluster shows a similar gene organization to its homologous in *S*. *avermitilis* [31]. It is organized in two divergent operons (*crtaEIBV* and *crtaQYTU*) that contain genes for the biosynthesis of carotenoid isorenieratene and biosynthesis intermediates [31] (Fig 1; S3 Table). In addition, it also contains *crtaT* and *crtaV* genes coding for a methyltransferase and a Rieske Non-Heme Iron-Dependent Oxygenase respectively, which have been also identified in other carotenoid BGCs. Noticeable in *S*. *griseus* these genes have been shown to be dispensable for the biosynthesis of isorenieratene, the end product of the carotenogenic pathway in this strain [34]. The *crta* BGC also contains a gene (*crtaQ*) coding for an extracytoplasmic sigma factor-like that is not found in the *crt* cluster of *S*. *avermitilis*.

*S*. *argillaceus* does not produce any carotenoid compound under standard laboratory conditions. It has been reported that actinomycetes can produce carotenoids in a light-induced manner or by the expression of an extracytoplasmic sigma factor [31]. Therefore, we tested if cultivation of *S*. *argillaceus* under light conditions or overexpressing the extracytoplasmic sigma factor-like *crtaQ* under the control of the erythromycin resistant promoter (pEM4T-sigmaC25) could activate production of carotenoid compounds in this microorganism. However, production of carotenoid compounds was absent in all conditions (data not shown). In *S*. *griseus* IFO13350 activation of a cryptic carotenoid gene cluster was achieved by increasing its copy number [35]. Therefore we envisioned to increase the copy number of the *crta* cluster to activate it. To do so, we identified a cosmid containing the entire *crta* cluster by screening a *S*. *argillaceus* cosmid library with PCR probes generated from the 5’- and 3’-ends of the *crta* cluster (fragment-I and fragment-D). In this way, cosmid pKC505-C25 was selected containing the whole *crta* cluster. This cosmid was expressed in *S*. *argillaceus* and in *S*. *albus* that do not produce any pigmented compound under standard laboratory conditions. As control, the empty vector pKC505 was also introduced in both strains. No differences were observed between *S*. *argillaceus*-pKC505-C25 and the control strain (data not shown). On the contrary, cultures of *S*. *albus*-pKC505-C25 became yellow pigmented while those from the control strain remained unpigmented, being highest production of the yellow pigment detected using SM17 media (Fig 3B). HPLC chromatograms of organic extracts from these cultures revealed several peaks with UV-vis spectra corresponding to carotenoid compounds (peaks **9** to **15**), which were absent in control cultures (Fig 3B). These carotenoids were identified by dereplication as 3,3’-dihydroxyleprotene (peak **9**), 3-hydroxyleprotene (peak **12**), leprotene (peak **14**) and β-isorenieratene (peak **15**) (Fig 2; S2 Fig). In addition, three new carotenoid compounds were detected that based on their molecular formula are proposed to be the monomethylated derivatives of 3,3’-dihydroxyleprotene (peak **10**) and 3-hydroxyleprotene (peak **13**), and the dimethylated derivative of 3,3’-dihydroxyleprotene (peak **11**), which is produced as the major compound (Figs 2 and 3B). The carotenogenic biosynthesis pathway of *S*. *argillaceus* would proceed through the synthesis of geranylgeranyl diphosphate units by CrtaE; formation of the C_40_ carbon chain phytoene by CrtaB; synthesis of lycopene through four desaturation steps by CrtaI; formation of β-carotene with two rings at both ends of the carbon chain by CrtaY; and synthesis of isorenieratene (leprotene) by a desaturation/methyltransferation mechanism in each ionone ring of β-carotene by CrtaU. Most of carotenogenic pathways in *Streptomyces* lead to isorenieratene as the final product [31,33], although in *S*. *mediolani* 3-hydroxy and 3,3’-dihydroxy derivatives of leprotene are the final products [36]. In the case of *S*. *argillaceus* this seems to be the dimethylated derivative of 3,3’-dihydroxyleprotene. Biosynthesis of this compound would require dihydroxylation of leprotene to generate 3,3’-dihydroxyleprotene, followed by its dimethylation. According to the genes identified in the *crta* cluster, candidate gene products to fulfill these functions could be the CrtaV Rieske Non-Heme Iron-Dependent Oxygenase and the CrtaT methyltransferase, respectively. 3,3’-dihydroxyleprotene was previously shown to have superior properties in preventing photo- and photooxidative damages in comparison to other carotenoids [37]. It remains to be determined if the three new compounds mono and dimethoxylated derivatives of leprotene here identified also show better properties.

### Inactivation of the global regulatory gene *adpAa* resulted in activation of germicidin gene cluster

**Fig 4:**
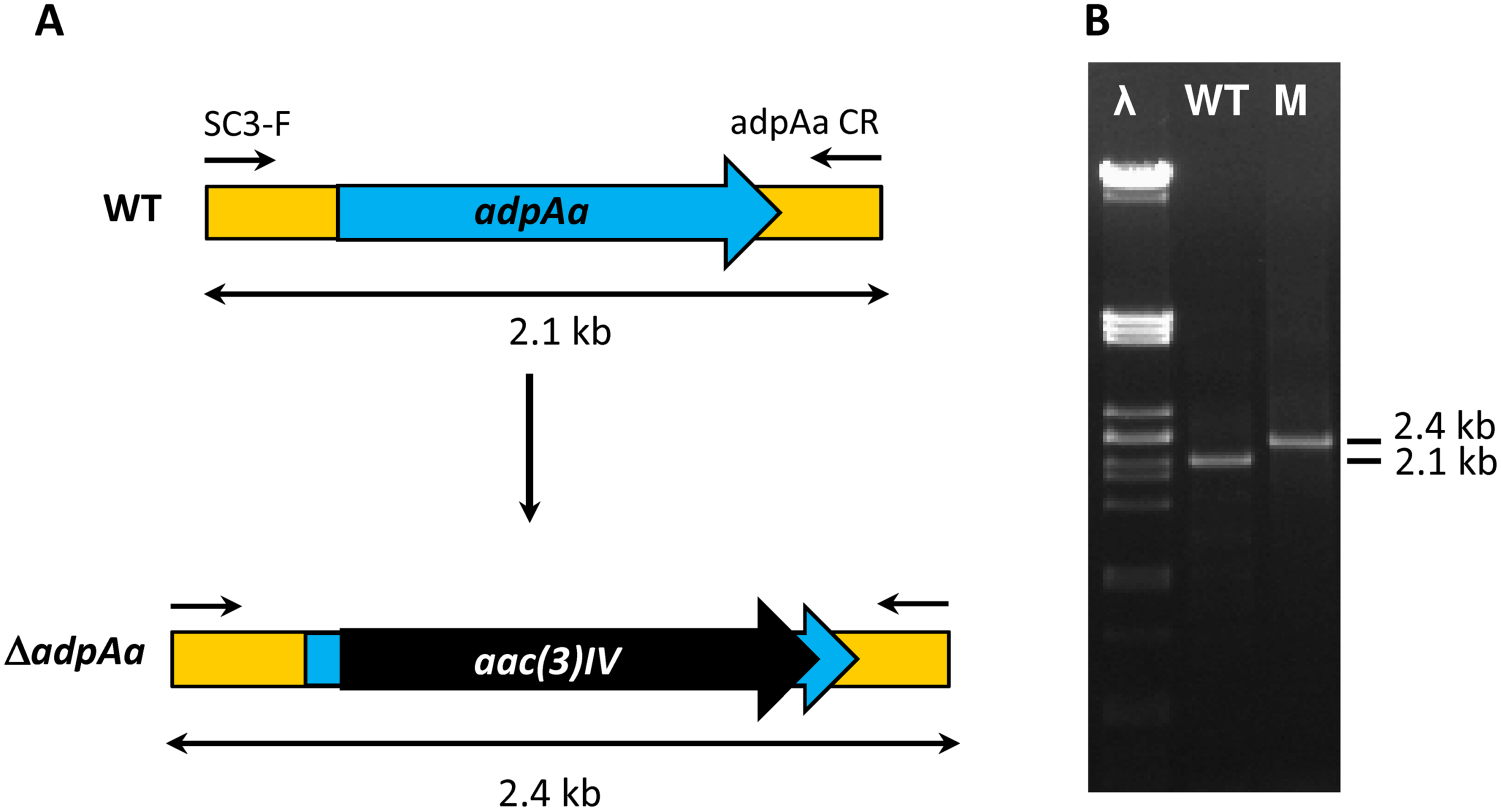
Generation of mutant *S*. *argillaceus* Δ*adpAa*. (A) Scheme representing the replacement event for generation of mutant Δ*adpAa*. WT, wild type strain; *aac(3)IV*, apramycin resistance gene; (B) PCR analysis of Δ*adpAa* mutant. PCR products from the wild type (WT) strain and from Δ*adpAa* mutant strain (M), using oligonucleotides SC3-F and adpAaCR. λ, Pst-digested Lambda DNA.

AdpA (A-factor-dependent protein A) is a key pleiotropic regulator first discovered in *S*. *griseus* where it acts as a positive transcriptional regulator of cell cycle differentiation and antibiotic production [38]. To determine the effect of inactivating *adpA* in *S*. *argillaceus*, it was necessary to clon that gene. Degenerated oligoprimers from conserved regions of *adpA* genes (S1 Table) were used to PCR amplified a homologous gene (*adpAa*) from *S*. *argillaceus*, which was later located in the *S*. *argillaceus* genome sequence [11] (nucleotides 2982405–2983677). This gene was mutated by replacing it by an apramycin resistance cassette that was inserted in the same direction of transcription than the target gene, generating mutant *S*. *argillaceus* Δ*adpAa*. The replacement event was confirmed by PCR (Fig 4): using oligonucleotides SCR-F and adpAa CR (S1 Table), a 2.1 kb DNA fragment was amplified from the wild type strain while a 2.4 kb was amplified from the mutant, confirming the replacement event.

Analysis of organic extracts of cultures of this mutant strain (*S*. *argillaceus* Δ*adpAa*) revealed that production of mithramycin strongly decreased (peak M, Fig 3C), but noticeable two compounds with similar retention times were accumulated (peaks **16** and **17**, Fig 3C). These compounds were not detected in the wild type strain (Fig 3C). Purification of each single compound was attempted but not achieved, being only possible to purify them as a mixture. Both compounds displayed a maximum at ca. 290 nm in the UV (DAD) spectrum and *m/z* values of 183 [M+H]^+^ what suggested being isomers. The HRMS information (S3 Fig) rendered a molecular formula of C_10_H_14_O_3_ based on the observed ion [M+H]^+^ at 183.1019 (calcd. for C_10_H_15_O_3_^+^ = 183.1016). Such formula and the observed UV (DAD) spectrum were compatible with germicidin B, isogermicidin B and germicidin C. The ^1^H NMR and the HSQC spectra (S4 Fig; S6 Table) revealed by comparison with reported data [39] that the sample comprises a mixture of germicidin B and germicidin C (Fig 2) in a ratio ca. 2.4:1. These compounds are known to have an inhibitory effect on germination of *Streptomyces* spores [40], and it has been proposed to play a role in coordinating the germination process within a spore population [41]. In *S*. *coelicolor* it has been shown that biosynthesis of germicidins only requires a Type III PKS (Gc) encoded by *sco7221*, in addition to enzymes from the fatty acid pathway to supply acyl-ACPs [39,42]. The AntiSMASH analysis of *S*. *argillaceus* genome did not identify those genes, but a deeper analysis of its genome allowed to identifying a gene (nucleotides 982748 to 983933) coding for a Type III PKS, which showed high similarity to germicidin synthases and was named *gcsA* (S4 Table). In addition, homologous genes to those involved in supplying acyl-ACPs (*fabD*, *fabH and acpA*) (Fig 1; S4 Table) were also identified in *S*. *argillaceus* genome, about 1.5 Mb upstream of *gcsA*. AdpA is considered a transcriptional activator of antibiotic production [38]. However, results shown here indicate that it can also play a negative role in production of specialized metabolites. Very recently it has been reported that a cryptic oviedomycin BGC in *S*. *ansochromogenes* was also activated by disrupting an *adpA* gene [43]. The activation of the germicidins production represents the second example of using *adpA*-inactivation as an approach to activate a silent pathway, and confirms this new strategy as a possible choice to be applied in genomics-driven discovery of natural products.

### Detection of desferrioxamine production by overexpressing the response regulator *abrC3*

AbrC3 is a response regulator that has been shown to positively control actinorhodin and undecylprodiginine production, and morphological differentiation in *S*.*coelicolor* [18]. Consequently, we tested the possibility of activating and/or enhancing production of specialized metabolites in *S*. *argillaceus* by overexpressing *abrC3*. After introducing *abrC3* (pHJLAbrC3c) into *S*. *argillaceus*, the recombinant strain was cultivated in R5A and SM17 media and cultures were extracted with ethyl acetate or butanol. Extracts were analyzed by HPLC-MS and compared with those obtained from cultures of a control strain containing the empty vector (pHJL401c). Cultures of *S*. *argillaceus*-pHJLAbrC3c in SM17 medium and extracted with butanol showed more than nine compounds that were either *de novo* produced or produced in higher amounts than in the control strain (Fig 3D). Dereplication of these samples allowed to identifying a compound that increased about 55% in relation to the control culture, eluted at 5.16 min and with an *m/z* value 561.3603 (Fig 3D; S5 Fig), for which the suggested molecular formula was C_25_H_48_N_6_O_8_ that corresponds to desferrioxamine B (Fig 2), a linear trihydroxamic acid siderophore with clinical application [44]. AntiSMASH analysis of *S*. *argillaceus* genome revealed that cluster 13 (*desa*; nucleotides 2970011–2977244) showed high similarity and synteny to the desferrioxamine gene cluster from *S*. *coelicolor* [45] (Fig 1; S5 Table). The *desa* cluster contains all genes required for the biosynthesis of desferrioxamine B [44] (S5 Table): a pyridoxal 5’-phosphate (PLP)-dependent lysine decarboxylase (*desaA*), a FAD-dependent monooxygenase (*desaB*), an acyl coenzyme A transferase (*desaC*) and a NTP-dependent siderophore synthetase (*desaD*). In addition, upstream of these four genes two genes are localized (*desaE* and *desaF*) that have been proposed to be involved in transporting cyclic trishydroxamate and utilization of iron from cyclic tris-hydroxamate iron-siderophore complexes, in *S*. *coelicolor* [45].

In conclusion, we have been able to activate four BGCs from *S*. *argillaceus*, proving their functionality. In addition, we have identified the putative encoded compounds. Further experiments (cluster inactivation and/or heterologous expression) would definitively proof the link between clusters *anta*, *gcs* and *desa* to the identified compounds. The selected BGCs are widely distributed among streptomycetes, but only in a few cases their functionality has been proved and the encoded compounds have been identified in cultures of those strains. The activation of these BGCs was achieved using different approaches: changing the cultivation conditions (for the antimycins *anta* cluster), expressing the cluster in a heterologous host (for the carotenoids *crta* cluster), and inactivating *adpAa* or overexpressing *abrC3* global regulatory genes (for the germicidins *gcsA* and desferrioxamine *desa* clusters, respectively). In addition, three new carotenoid compounds have been identified. Results shown here will contribute to extend the knowledge about the specialized metabolism of the *S*. *argillaceus* strain, which could be used to activate and identify other specialized metabolites and to improve and generate new compounds by metabolic engineering.

## Supporting information

S1 Fig HPLC-MS analyses of compounds in peaks identified in Fig 3A. (DOCX)

S2 Fig MS analyses of compounds in peaks identified in Fig 3B. (DOCX)

S3 Fig HRMS spectra of germicidins. (DOCX)

S4 Fig ^1^H NMR and HSQC spectra of germicidins (CD_Cl3_, 500 MHz). (DOCX)

S5 Fig MS analysis of compound in peak 18 in Fig 3D. (DOCX)

S1 File Composition of production media. (DOCX)

S1 Table Oligonucleotides used in this work. (DOCX)

S2 Table Functions of gene products for antimycin gene cluster (*anta*). (DOCX)

S3 Table Functions of gene products for isorenieratene gene cluster (*crta*). (DOCX)

S4 Table Functions of gene products for germicidin gene cluster (*gcsA*). (DOCX)

S5 Table Functions of gene products for desferrioxamine gene cluster (*desa*). (DOCX)

S6 Table NMR data of germicidins B and C. (DOCX)
