# Supplementary material for: Uncovering production of specialized metabolites by Streptomyces argillaceus: Activation of cryptic biosynthesis gene clusters using nutritional and genetic approaches
Source: PLoS One. 2018 May 24;13(5):e0198145. doi: 10.1371/journal.pone.0198145 (PMC5993118; doi:10.1371/journal.pone.0198145)
Supplement: S1 Fig — (DOCX) [file pone.0198145.s001.docx]

**S1 Fig. HPLC-MS analyses of compounds in peaks identified in Fig 3A**: (A) peaks **1** and **2** (antimycin A_4_), (B) **3** and **4** (antimycin A_3_,), (C) **5** and **6** (antimycin A_2_), and (D) **7** and **8** (antimycin A_1_).
