# Supplementary material for: Uncovering production of specialized metabolites by Streptomyces argillaceus: Activation of cryptic biosynthesis gene clusters using nutritional and genetic approaches
Source: PLoS One. 2018 May 24;13(5):e0198145. doi: 10.1371/journal.pone.0198145 (PMC5993118; doi:10.1371/journal.pone.0198145)
Supplement: S2 Fig — (DOCX) [file pone.0198145.s002.docx]

**S2 Fig. MS analyses of compounds in peaks identified in Fig 3B**: (A) peak **9** (3,3’-dihydroxyleprotene); (B) peak **10** (3-methoxy-3’-hydoxyleprotene); (C) peak **11** (3,3’-dimethoxyleprotene); (D) peak **12** (3-hydroxyleprotene); (E) peak **13** (3-methoxyleprotene); (F) peak **14** (leprotene); and (G) peak **15** (β-isorenieratene).
