# Supplementary figures and images for: Uncovering production of specialized metabolites by Streptomyces argillaceus: Activation of cryptic biosynthesis gene clusters using nutritional and genetic approaches
Source: PLoS One. 2018 May 24;13(5):e0198145. doi: 10.1371/journal.pone.0198145 (PMC5993118; doi:10.1371/journal.pone.0198145)

**S3 Fig. HRMS spectra of germicidins**


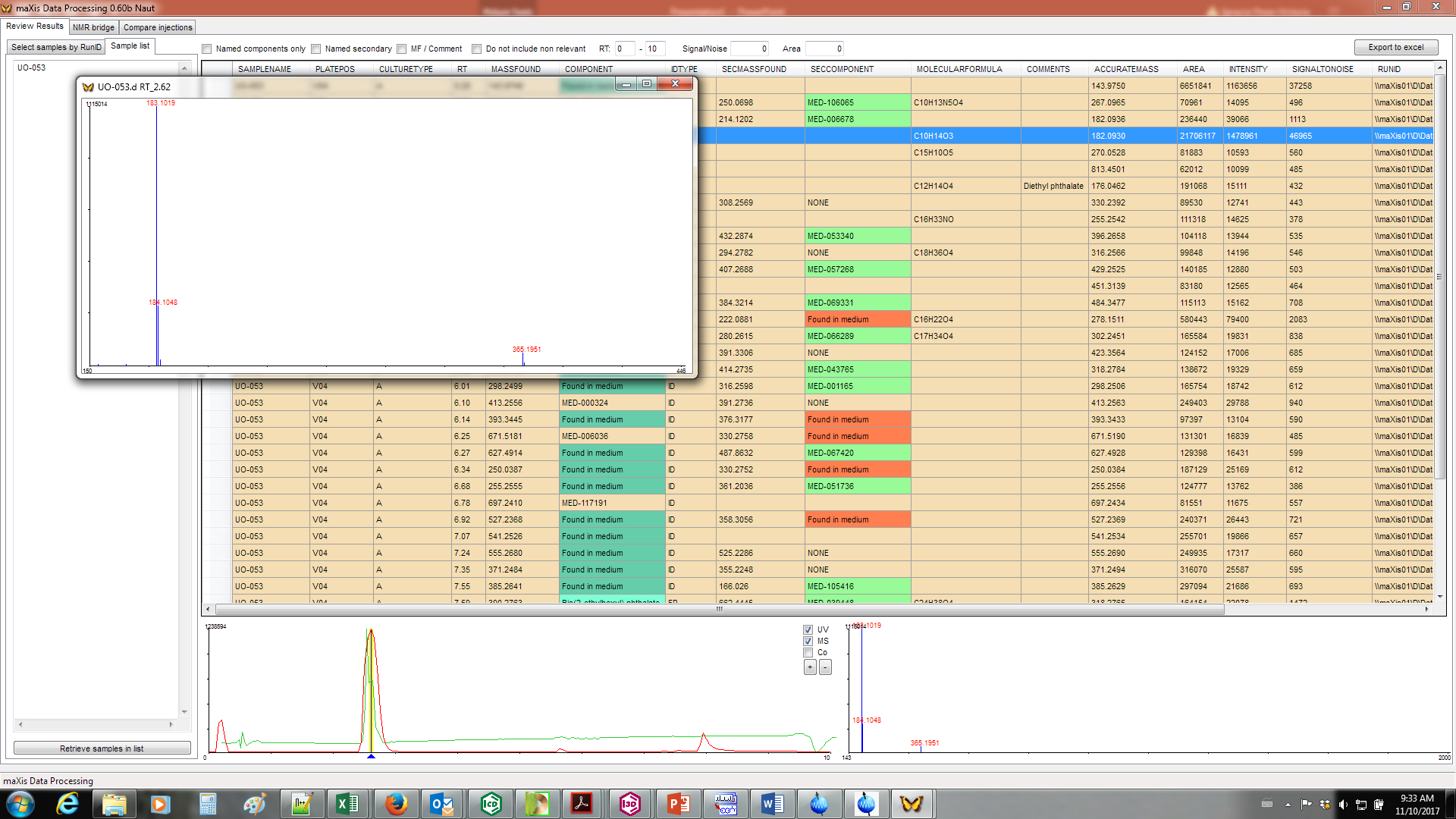

Supplement: S3 Fig — (DOCX) [file pone.0198145.s003.docx]

**S4 Fig. 1H NMR (upper) and HSQC (lower) spectra of germicidins (CDCl3, 500 MHz)**


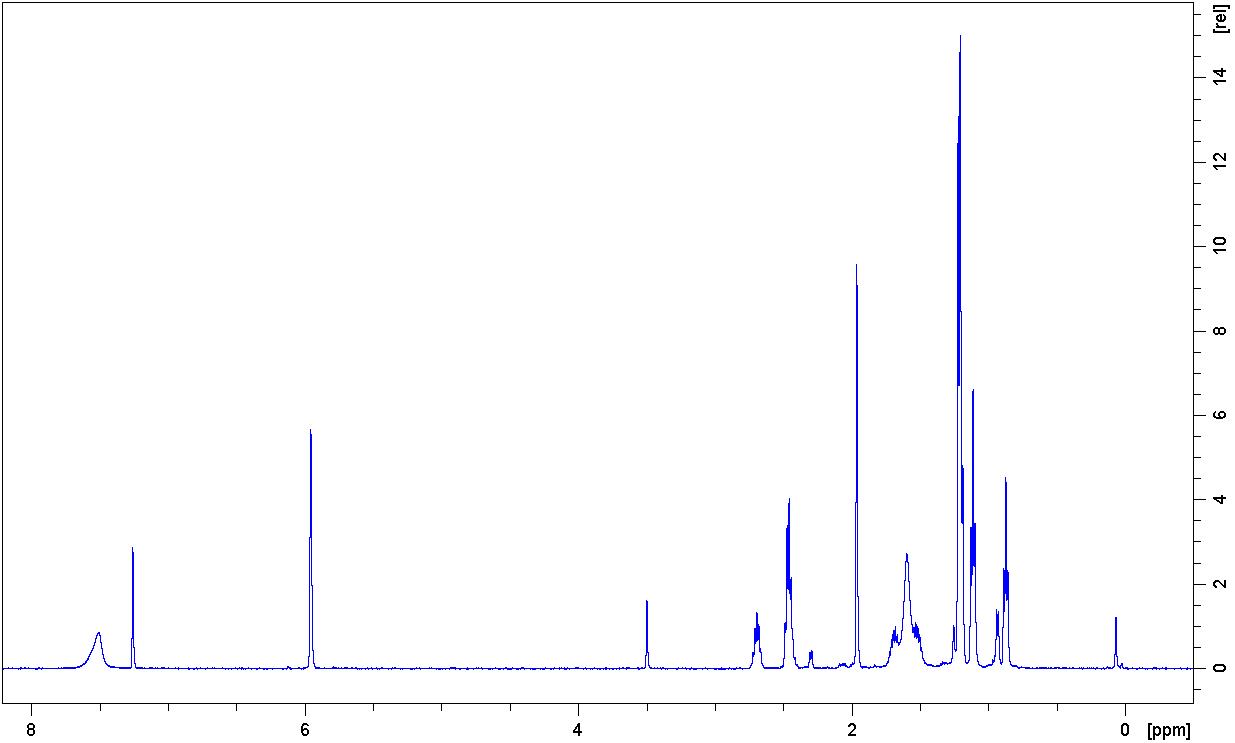


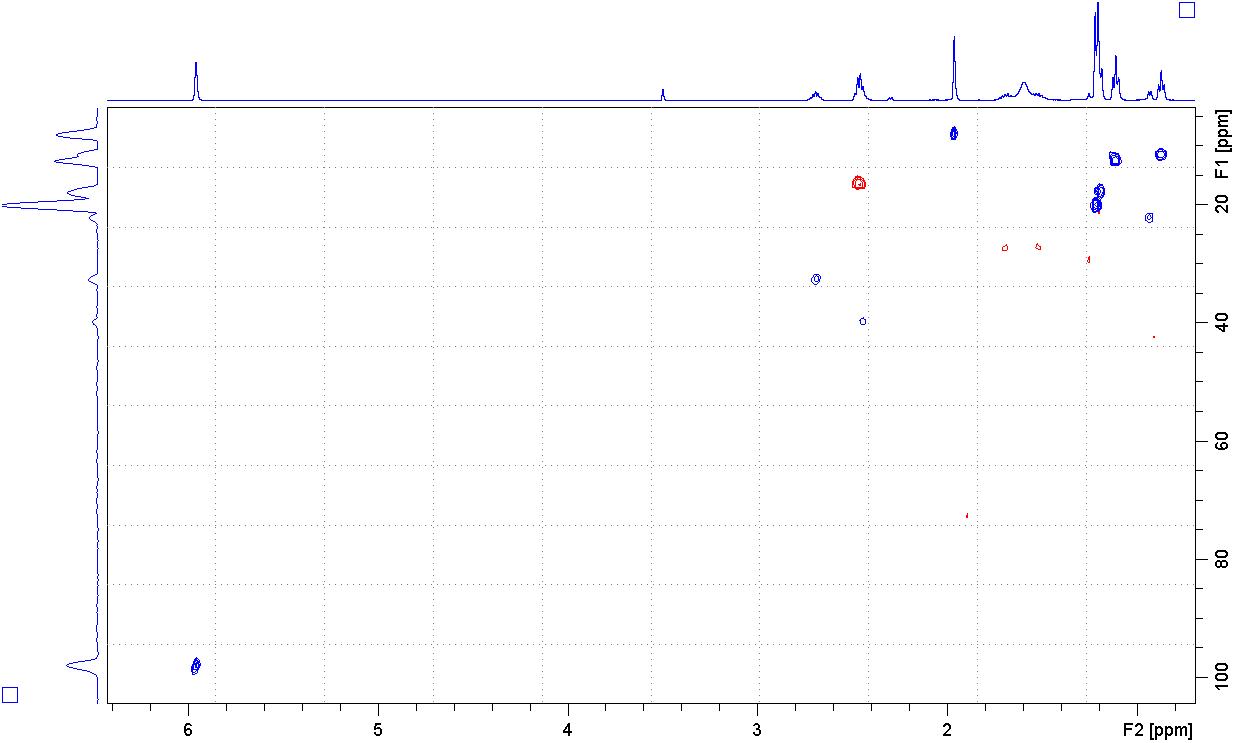

Supplement: S4 Fig — (DOCX) [file pone.0198145.s004.docx]
