# Supplementary material for: Uncovering production of specialized metabolites by Streptomyces argillaceus: Activation of cryptic biosynthesis gene clusters using nutritional and genetic approaches
Source: PLoS One. 2018 May 24;13(5):e0198145. doi: 10.1371/journal.pone.0198145 (PMC5993118; doi:10.1371/journal.pone.0198145)
Supplement: S5 Fig — (DOCX) [file pone.0198145.s005.docx]

**S5 Fig**. **MS analysis of compound in peak 18 in Fig 3D**

**561.3603**
