# Supplementary material for: Uncovering production of specialized metabolites by Streptomyces argillaceus: Activation of cryptic biosynthesis gene clusters using nutritional and genetic approaches
Source: PLoS One. 2018 May 24;13(5):e0198145. doi: 10.1371/journal.pone.0198145 (PMC5993118; doi:10.1371/journal.pone.0198145)
Supplement: S1 File — (DOCX) [file pone.0198145.s006.docx]

**S1 File. Composition of production media**

SM3: glucose, 5 g/l; maltodextrin, 50 g/l; soy protein, 25 g/l; syrup, 3 g/l; K_2_HPO_4_, 0.25 g/l; CaCO_3_, 2.5 g/l; pH 7.0.

SM4: glucose, 2.5 g/l; maltodextrin, 25 g/l; soy protein, 12.5 g/l; syrup, 1.5 g/l; K_2_HPO_4_, 0.125 g/l; CaCO_3_, 1.25 g/l; MOPS, 21 g/l; pH 6.5.

SM19: tomato paste, 40 g/l; oat flour, 15 g/l; glucose, 2 g/l; tap water.

SM30: tomato paste, 40 g/l; oat flour, 15 g/l; glucose, 2 g/l; tap water; pH 4.5.

SV2: glucose, 15 g/l; glycerol, 15 g/l; soy peptone, 15 g/l; NaCl, 3 g/l; CaCO_3_, 1 g/l; pH 7.0.
