# Supplementary material for: Uncovering production of specialized metabolites by Streptomyces argillaceus: Activation of cryptic biosynthesis gene clusters using nutritional and genetic approaches
Source: PLoS One. 2018 May 24;13(5):e0198145. doi: 10.1371/journal.pone.0198145 (PMC5993118; doi:10.1371/journal.pone.0198145)
Supplement: S1 Table — (DOCX) [file pone.0198145.s007.docx]

**S1 Table. Oligonucleotides used in this work**

| **PRIMER** | **SEQUENCE 5’-3’** |
| --- | --- |
| sigmaC25 up | ATAGGATCCCGGACCCGGACGAGAAG |
| sigmaC25 rp | ATAGAATCCAGCGGGGTCGCATCGAT |
| genot25I up | GGTGTCGTGTGCGTCATGCC |
| genot25I rp | TGATCGGGCTGCAGATGCTG |
| genot25D up | CTACGGCTCCCTCGACAACG |
| genot25D rp | GTTGAGCGACGACAGCAGGA |
| adpA F | TCGTYGCSGTGCTGCTGTTCAGCG |
| adpA R | GCGTCTCCACGTCGAACTGCTCGT |
| SC3-F | CTACTAGTCGTGCAGGAGCGACACAA |
| adpAa CR | GCAGTTGACTTTGTGGTCT |
| adpA 3SpeI | GACTAGTCCAAGTGCTTCACCAACACCA |
| adpA 3NsiI | CGCATGCATGGAATGGAACTCTCGAAGAT |
| adpA 5BglII | GTAGATCTCGTCACCCTTAGGGTGGA |
| adpA 2R | GTGATATCCCGTCGAGCACCTGCT |
