# Supplementary material for: Uncovering production of specialized metabolites by Streptomyces argillaceus: Activation of cryptic biosynthesis gene clusters using nutritional and genetic approaches
Source: PLoS One. 2018 May 24;13(5):e0198145. doi: 10.1371/journal.pone.0198145 (PMC5993118; doi:10.1371/journal.pone.0198145)
Supplement: S2 Table — (DOCX) [file pone.0198145.s008.docx]

**S2 Table. Functions of gene products from antimycin gene cluster (*anta*)**

| **Gene** | **Size (aa)** | **Proposed function** | **Similar protein (acc. number)** | **Identical aa (%)** |
| --- | --- | --- | --- | --- |
| *antaA* | 172 | RNA polymerase sigma factor | WP_030823993.1 | 91 |
| *antaB* | 454 | putative acyltransferase | WP_094102420.1 | 83 |
| *antaC* | 2933 | non-ribosomal peptide synthetase | WP_053636314.1 | 84 |
| *antaD* | 1271 | type I polyketide synthase | WP_030864709.1 | 87 |
| *antaE* | 409 | crotonyl-CoA reductase | WP_037711109.1 | 93 |
| *antaF* | 494 | acyl-CoA ligase | WP_073497929.1 | 95 |
| *antaG* | 86 | acyl carrier protein | WP_046248796.1 | 90 |
| *antaH* | 404 | phenylacetate-CoA oxygenase | WP_037637554.1 | 94 |
| *antaI* | 103 | 1,2-phenylacetyl-CoA epoxidase | WP_073497933.1 | 94 |
| *antaJ* | 285 | phenylacetate-CoA oxygenase | WP_094102373.1 | 85 |
| *antaK* | 170 | phenylacetate-CoA oxygenase | WP_030864683.1 | 82 |
| *antaL* | 399 | phenylacetate-CoA oxygenase | OQD57828.1 | 85 |
| *antaM* | 263 | 3-oxoacyl-ACP reductase | WP_094102372.1 | 95 |
| *antaN* | 282 | tryptophan 2,3-dioxygenase | WP_073497937.1 | 92 |
| *antaO* | 282 | lipase | WP_086733553.1 | 91 |
| *antaP* | 419 | kynureninase | WP_094102371.1 | 92 |
