# Supplementary material for: Uncovering production of specialized metabolites by Streptomyces argillaceus: Activation of cryptic biosynthesis gene clusters using nutritional and genetic approaches
Source: PLoS One. 2018 May 24;13(5):e0198145. doi: 10.1371/journal.pone.0198145 (PMC5993118; doi:10.1371/journal.pone.0198145)
Supplement: S3 Table — (DOCX) [file pone.0198145.s009.docx]

**S3 Table. Functions of gene products from isorenieratene gene cluster (*crta*)**

| **Gene** | **Size (aa)** | **Proposed function** | **Similar protein (acc. number)** | **Identical aa (%)** |
| --- | --- | --- | --- | --- |
| *crtaV* | 338 | 2Fe-2S ferredoxin | WP_059193421.1 | 82 |
| *crtaB* | 343 | phytoene/squalene synthase | WP_059078035.1 | 81 |
| *crtaI* | 514 | phytoene desaturase | WP_045561225.1 | 90 |
| *crtaE* | 463 | geranylgeranyl pyrophosphate synthase | WP_060889439.1 | 77 |
| *crtaQ* | 206 | sigma factor | WP_069767876.1 | 78 |
| *crtaY* | 396 | lycopene cyclase | WP_060905755.1 | 77 |
| *crtaT* | 244 | methyltransferase | WP_078631810.1 | 82 |
| *crtaU* | 542 | isorenieratene synthase | WP_057577082.1 | 87 |
