# Supplementary material for: Uncovering production of specialized metabolites by Streptomyces argillaceus: Activation of cryptic biosynthesis gene clusters using nutritional and genetic approaches
Source: PLoS One. 2018 May 24;13(5):e0198145. doi: 10.1371/journal.pone.0198145 (PMC5993118; doi:10.1371/journal.pone.0198145)
Supplement: S4 Table — (DOCX) [file pone.0198145.s010.docx]

**S4 Table. Functions of gene products from germicidin gene cluster (*gcsA*)**

| **Gene** | **Size (aa)** | **Proposed function** | **Similar protein (acc. number)** | **Identical aa (%)** |
| --- | --- | --- | --- | --- |
| *gcsA* | 394 | germicidin synthase | SOE09805.1 | 92 |
| *fabD* | 304 | ACP S-malonyltransferase | WP_086770639.1 | 94 |
| *fabH* | 333 | ketoacyl-ACP synthase III | WP_073493234.1 | 98 |
| *acpA* | 82 | acyl carrier protein | WP_031131297.1 | 99 |
