# Supplementary material for: Uncovering production of specialized metabolites by Streptomyces argillaceus: Activation of cryptic biosynthesis gene clusters using nutritional and genetic approaches
Source: PLoS One. 2018 May 24;13(5):e0198145. doi: 10.1371/journal.pone.0198145 (PMC5993118; doi:10.1371/journal.pone.0198145)
Supplement: S5 Table — (DOCX) [file pone.0198145.s011.docx]

**S5 Table. Functions of gene products for desferrioxamine gene cluster (*desa*)**

| **Gene** | **Size (aa)** | **Proposed function** | **Similar protein (acc. number)** | **Identical aa (%)** |
| --- | --- | --- | --- | --- |
| *desaE* | 349 | ABC transporter substrate-binding protein | WP_073491960.1 | 92 |
| *desaF* | 185 | siderophore-interacting protein | WP_094102948.1 | 84 |
| *desaA* | 480 | pyridoxal 5’-phosphate (PLP)-dependent lysine decarboxylase | WP_073491961.1 | 95 |
| *desaB* | 430 | FAD-dependent monooxigenase | WP_004987747.1 | 90 |
| *desaC* | 183 | N-acetyltransferase | WP_079274926.1 | 88 |
| *desaD* | 599 | NTP-dependent siderophore synthetase | WP_073491963.1 | 90 |
