# Supplementary material for: Uncovering production of specialized metabolites by Streptomyces argillaceus: Activation of cryptic biosynthesis gene clusters using nutritional and genetic approaches
Source: PLoS One. 2018 May 24;13(5):e0198145. doi: 10.1371/journal.pone.0198145 (PMC5993118; doi:10.1371/journal.pone.0198145)
Supplement: S6 Table — (DOCX) [file pone.0198145.s012.docx]

**S6 Table. NMR data of germicidins B and C**

| **Germicidin B** | | |  | **Germicidin C** | | |
| --- | --- | --- | --- | --- | --- | --- |
| position | δ_H_, m (*J* in Hz*)* | δ_C_, type |  | position | δ_H_, m (*J* in Hz*)* | δ_C_, type |
|  |  |  |  |  |  |  |
| 2 | - | n. d. |  | 2 | - | n. d. |
| 3 | - | n. d. |  | 3 | - | n. d. |
| 4 | - | n. d. |  | 4 | - | n. d. |
| 5 | 5.97, s | 97.9, CH |  | 5 | 5.97, s | 97.9, CH |
| 6 | - | n. d. |  | 6 | - | n. d. |
| 1’ | 2.47, m | 16.5, CH_2_ |  | 1’ | 1.97, s | 7.9, CH_3_ |
| 2’ | 1.13, t (7.3) | 12.5, CH_3_ |  | 1’’ | 2.45, m | 39.7, CH |
| 1’’ | 2.70, sep (6.8) | 32.4, CH |  | 2’’ | 1.70, m  1.53, m | 27.3, CH_2_ |
| 2’’ | 1.23, m | 19.9, CH_3_ |  | 3’’ | 0.89, t (7.3) | 11.4, CH_3_ |
| 3’’ | 1.23, m | 19.9, CH_3_ |  | 4’’ | 1.21, m | 17.7 |

^13^C NMR data derived from HSQC spectra

Germicidins B (left) and C (right)
